# Incidence of malnutrition and changes in phosphocalcic metabolism in perioperative liver transplantation patients -a retrospective study in a tertiary children’s hospital in China

**DOI:** 10.1186/s12887-022-03790-5

**Published:** 2022-12-15

**Authors:** Liya Pan, Xuejie Gao, Huanhuan Fu, Yunman Liu, Li Hong

**Affiliations:** 1 Department of Clinical Nutrition, Shanghai Children’s Medical Center/National Children’s Medical Center, School of Medicine, Shanghai Jiao Tong University School of Medicine, 1678 Dongfang Road, Shanghai, 200127 China; 2 Department of Gastroenterology, Fujian Maternity and Child Health Hospital College of Clinical Medicine for Obstetrics and Gynecology and Pediatrics, Fujian Medical University, Fujian, China

**Keywords:** Liver transplantation, Dual energy X-ray absorptiometry, Bone mineral density, Vitamin D

## Abstract

**Background:**

The primary aim of the study was to assess the nutritional status of pediatric liver transplant outpatients in nutrition clinic, particularly the nutritional status of their bones.

**Methods:**

One hundred thirty-eight pediatric liver transplant outpatients, who had visited the nutrition clinic in Shanghai Children’s Medical Center between January 2017 and December 2019, were recruited. The bone mineral density (BMD) z-scores were determined by dual energy X-ray absorptiometry (DXA). Nutritional assessment was performed, and their corresponding height-for-age z-scores (HAZs)/weight-for-age z-scores (WAZs)/BMI-for-age z-scores (BMIZs) were obtained.

**Results:**

A total of 138 patients came to our nutrition outpatient clinic, including 68 boys (49.3%) and 70 girls (50.7%). The median age was 0.87y (0.68y, 1.71y). Among these patients, 44 (31.9%) had acute malnutrition with WAZ/BMIZ value -1.14 (-2.38, -0.18), 55 (38.4%) had chronic malnutrition with HAZ value -1.51 (-2.39, -0.38), and 96 (69.6%) had a BMD lower than normal. The BMD z-score was significantly correlated with the WAZ/BMIZ value (Spearman’s correlation coefficient = 0.334, *p* < 0.001). A total of 37 infants re-visited the nutrition clinic for a follow-up after (147 ± 127) days. The WAZ/BMIZ value of the re-visiting patients and the BMD z-score of the re-visiting patients were significantly improved compared to those of the first-visit patients (*p* = 0.004 and *p* = 0.001 respectively).

**Conclusions:**

There were different rates of malnutrition before and after liver transplantation. At the same time, BMD Z-score and serum vitamin D level of patients decreased. There was a significant correlation between BMD z-scores and WAZ/BMIZ values. Proper and professional nutrition guidance significantly improved the WAZ/BMIZ-values and BMD Z-score of liver transplantation patients.

## Background

The liver is the largest and the most important metabolic organ of human body. It plays a key role in the metabolism of various macronutrients and micronutrients [1]. With the continuous development of surgical technology in the field of liver transplantation, liver transplantation has become the optimal treatment for pediatric patients with end-stage liver disease, liver failure, liver-based genetic metabolic diseases and liver tumors that cannot be treated with conventional treatments [2, 3]. The 1-year survival rate of pediatric liver transplant patients could reach 90%, and the 15–20-year survival rate of them could reach 75% [4]. Therefore, long-term maintenance of life quality after liver transplantation has become more and more significant.

The nutritional status of patients is a relevant factor to determine the progression of liver disease. Metabolic disorders, inadequate nutrients intake, absorption disorders, and a hypermetabolic status may lead to malnutrition in patients during the perioperative period of liver transplantation, increase the surgical risk, increase the incidence of postoperative complications, prolong the length of hospital stay, increase the cost of hospitalization, and affect the prognosis of patients after liver transplantation [5, 6].

Bone nutrition status of patients are closely related to the malnutrition. Bone mass loss is common in liver transplant patients [7, 8]. Bone mineral density (BMD) measurement is one of the key methods for the diagnosis of nutritional status of bones. Currently being recognized as the “gold standard” for the diagnosis of osteoporosis [9], the dual energy X-ray absorptiometry (DXA) can eliminate the effects of surrounding soft tissues and bone fats on the measurement results.

The vitamin D level is another parameter reflecting the nutrition status of bone. Vitamin D is a type of fat-soluble ring opening steroid. It has extra skeletal effects on body’s muscles, cardiovascular system, metabolism, immunity, tumorigenesis, pregnancy and fetal development [10]. By mitigating the glucocorticoid induced reduction of intestinal calcium absorption, active vitamin D3 can alleviate the secondary hyperparathyroidism of parathyroid glands, promote the differentiation and maturation of osteoblast precursors, enhance the immunosuppressive effect of cyclosporine A, and reduce bone loss after liver transplantation [11, 12]. Even in healthy children, vitamin D deficiency is very common. China and other countries have all issued domestic and international guidelines for the prevention and treatment of vitD deficiency [13, 14].

This study aimed to assess the nutritional status of pediatric liver transplant outpatients in nutrition clinic, and to assess the nutritional status of liver transplant patients’ bones based on serum 1,25-(OH)_2_-VitD3 and bone mineral density (BMD) measured by dual energy X-ray absorptiometry (DXA). We also aimed to analyze the relationship between patients’ BMD results and WAZ/HAZ/BMIZ values, and to observe patients’ nutritional status after nutritional intervention, including any improvement of nutritional status of patients’ bones. Our ultimate purpose was to define the significance of nutritional intervention in improving the nutritional status of liver transplant infants, particularly the nutritional status of their bones.

## Methods

### Research subject and data collection

It was a retrospective study. One hundred thirty-eight pediatric liver transplant outpatients, who went to the nutrition clinic in Shanghai Children’s Medical Center between January 2017 and December 2019, were selected as study subjects. Because the aim of our study was analyzing the nutritional status and bone mineral density of children undergoing liver transplantation, children in the peri-liver transplantation period who visited the nutrition department and were willing to accept bone mineral density testing were included in the study.

Patients’ basic information including gender, height, weight, primary disease, date of liver transplantation and feeding status were collected.

Young children were weighed on a lying-down scale, and children able to stand without assistance were weighed on an upright scale. Length/height measurements were obtained from using a tape measure for young children and a height scale for older children.

The BMD of the infants was measured and the BMD z-score was determined by DXA (DXA-3000, Shanghai Osteosys Co., Ltd.). A z-score equal to or greater than zero indicated a normal BMD. Serum calcium, serum 1,25-(OH)_2_-VitD3, serum alanine aminotransferase, and serum AKP levels were also measured.

The study was performed according to the criteria of the Helsinki II Declaration. All procedures involving human subjects/patients were approved by the Ethics Committee of Shanghai Children’s Medical Center. Participants completed a written informed consent form prior to being enrolled in the study.

### Nutritional assessment and intervention

The height and weight of outpatients who visited our nutrition clinic were measured. The nutritional status of patients aged 0–2 was assessed according to HAZs/WAZs, and that of patients aged greater than 2 was assessed according to BMIZ values.

An application (version 3.2.2) provided by WHO for height/weight/BMI measurements was downloaded from http://www.who.int/childgrowth/software/en/. It was used to assess patients’ nutritional status according to their height/weight/BMI-for-age/HAZ/WAZ/BMIZ values. A WAZ/BMI z-score lower than -2 was defined as acute malnutrition, and a HAZ lower than -2 was defined as chronic malnutrition [15]. We also carefully asked about patients’ nutritional intake, particularly the intake of vitD. Following base-line data collection and analysis of nutritional status, individualized diets were made for patients based on their target nutrition intake, dietary habits and current diets. Formulas high in calorie and MCT were delivered to increase patients’ intake of calories and various nutrients, so as to improve patients’ nutritional status. The energy intake was recommended to be 110%-150% RNI. Nutrients in the diets were distributed as follows: 12%-16% total kcal was from protein, 30%-40% was from fat, and 40%-45% was from carbohydrate. We recommended patients to take high-quality protein and high MCT formulas. Patients after liver transplantation should also pay careful attention to the supplementation of water-soluble vitamins and minerals, including selenium, zinc, calcium, and magnesium. We recommended a supplementation of 800–1000 IU/d of vitD for patients with negative BMD z-scores, and a supplementation of 400–800 IU/d of vitD for patients with zero or positive BMD z-scores.

### Follow-up visit

Patients were recommended for a follow-up visit to our nutrition outpatient clinic in 1–2 months. We re-measured children’s height, weight, BMD, and AKP levels during their follow-up visit.

### Statistical analysis

SPSS 16.0 software was used for statistical analysis. All the variables were compared for normal distribution using the Kolmogorov–Smirnov test. Categorical variables were expressed as number and percentage and analyzed with the chi square test, while continuous variables were expressed as mean ± standard deviation or median (interquartile range) as indicated. Wilcoxon test was used for the comparison of data of the same category. Mann Whitney rank sum test was applied for the comparison of BMD z-scores between those of malnourished and non-malnourished infants. Spearman correlation analysis and cross table analysis were used for determining the relationship between BMD z-scores and nutritional status z-scores, and that between BMD z-scores and AKP levels. A *p* value < 0.05 was considered statistically significant.

## Results

### General and baseline characteristics of patients

A total of one hundred thirty-eight children with liver transplantation in perioperative period visited our nutrition outpatient clinic, and their BMD were measured. Among 138 pediatric patients, 68 were boys (49.3%) and 70 were girls (50.7%). The median age of the patients was 0.87y (0.68y, 1.71y), of which 113 (81.9%) were under 2 years old and 25 (18.1%) were over 2 years old. The study population included 13 patients (9.4%) before liver transplantation, 80 patients (58.0%) within 100 days after transplantation, and 45 patients (32.6%) more than 100 days after transplantation. 115 patients’ primary disease was biliary atresia. All the above information was shown in Table 1.

**Table 1:** General and baseline characteristics of pediatric patients with liver transplantation

Clinical Status	
Gender (n,%)	
Boys	68 (49.3%)
Girls	70 (50.7%)
Age, Median (IQR)	0.87y (0.68y, 1.71y)
Primary disease (n, %)	
Ornithine aminotransferase deficiency	1 (0.7%)
Progressive familial intrahepatic cholestasis	1 (0.7%)
Wilson’s disease	1 (0.7%)
Alagille Syndrome	4 (2.9%)
Propionic acidemia	1 (0.7%)
Biliary atresia	115 (83.4%)
Cholestasis	3 (2.3%)
Hepatoblastoma	2 (1.5%)
Liver cirrhosis	1 (0.7%)
Methylmalonic acidemia	4 (2.9%)
Antitrypsin deficiency	1 (0.7%)
Langerhans cell histiocytosis	1 (0.7%)
Tyrosinemia	1 (0.7%)
Cavernous transformation of portal vein	1 (0.7%)
Niemann-Pick disease	1 (0.7%)
At the clinic visit, had liver transplantation or not (n, %)	
Before liver transplantation	13 (9.4%)
Within 100 days after transplantation	80 (58.0%)
More than 100 days after transplantation (including 100 days)	45 (32.6%)

### Nutritional status assessment and BMD measurements

Patients’ nutritional status was assessed based on their WAZ, HAZ, and BMIZ values. As mentioned in ‘method’ part, A WAZ/BMI z-score lower than -2 was defined as acute malnutrition, and a HAZ lower than -2 was defined as chronic malnutrition. Forty-four patients (31.9%) had acute malnutrition, and fifty-three patients (38.4%) had chronic malnutrition. Additionally, WAZ/BMIZ-values, HAZ-values, BMD z-values and vitamin D levels, liver functions index were shown in Table 2.

**Table 2:** Changes of anthropometric parameters, BMD levels, and serum indexes in patients’ first and follow-up visits

	At first visit, median (IQR)	At follow-up visit, median (IQR)	*p*-value
WAZ/BMIZ-values	-1.14 (-2.38, -0.18)	-0.80 (-1.81, 0.23)	0.004
HAZ values	-1.51 (-2.39, -0.38)	-1.52 (-2.20, -0.17)	0.474
BMD z-values	-0.55 (-1.00, 0.10)	-0.10 (-0.70, 0.70)	0.001
1,25-(OH)_2_-D3(ng/ml)	23.21 (19.65, 29.11)	/	
Serum calcium levels (mmol/L)	1.70 (1.60, 1.75)	/	
Serum ALT levels(IU/L)	35.00 (26.00, 53.00)	43.50 (28.00, 67.25)	0.577
Serum AKP levels (IU/L)	256.00 (192.00, 334.75)	305.00 (240.50, 366.25)	0.002

Abbreviations: *WAZ/BMIZ* weight-for-age z-scores/BMI-for-age z-scores, *HAZ* height-for-age z-scores, *BMD* bone mineral density

**Fig. 1:**
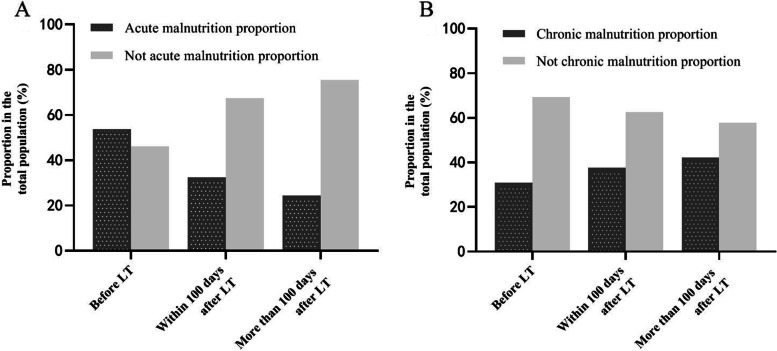
Comparison of the proportions of acute and chronic malnourished patients in different groups when visited our nutrition outpatient clinic. LT: liver transplantation. **A** A comparison of the proportion of acute malnourished patients in different groups when visited our nutrition outpatient clinic. **B** A comparison of the proportion of chronic malnourished patients in different groups when visited our nutrition outpatient clinic

Patients were grouped according to whether they already had liver transplantation or not when they visited the nutrition outpatient clinic. Among 13 patients who visited our nutrition outpatient clinic before liver transplantation, 7 (53.8%) had acute malnutrition, and 4 (30.8%) had chronic malnutrition. Among patients who visited our nutrition outpatient clinic within 100 days after liver transplantation, 26 (32.5%) had acute malnutrition, and 30 (37.5%) had chronic malnutrition. Among patients who visited our nutrition outpatient clinic more than 100 days after liver transplantation, 11 (24.4%) had acute malnutrition, and 19 (42.2%) had chronic malnutrition (Fig. 1). There were no statistically significant differences between different groups of the days patients after liver transplantation and the proportion of acute/chronic malnutrition. (*p* = 0.062 and 0.435 respectively by chi square test).

**Fig. 2:**
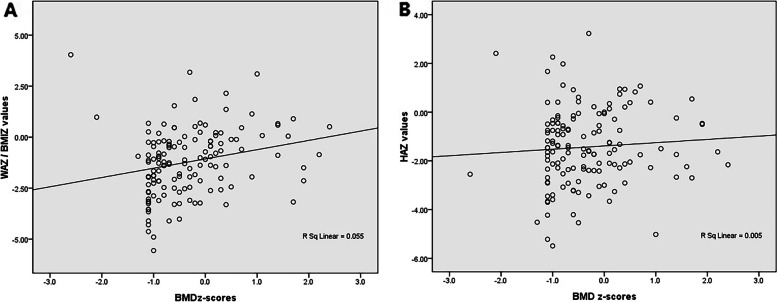
The correlation analysis between BMD z-values and anthropometric z-values. **A** There was a significant positive relationship between BMD z-values and WAZ/BMIZ values (Spearman correlation coefficient = 0.334, *p* < 0.001). **B** There was no significant relationship between BMD z-values and HAZ values (Spearman correlation coefficient = 0.131, *p* = 0.128)

Ninety-six patients (69.6%) had a BMD z-score lower than 0, and 42 patients (30.4%) had a BMD z ≥ 0. The median BMD z-score of patients was -0.55 (-1.00, 0.10). For patients who visited our nutrition outpatient clinic before liver transplantation, the median BMD z-score was -0.80 (-1.00, 0.15). The median BMD z-score of patients who visited nutrition clinic within 100 days after liver transplantation was -0.70 (-1.00, 0.10), and that of patients who visited nutrition clinic more than 100 days after liver transplantation was -0.40 (-0.85, 0.20). Spearman correlation analysis was used to analyze the relationship between patients’ BMD results and WAZ /BMIZ/HAZ values. As a result, there was a significant correlation between BMD z-scores and WAZ/BMIZ values (Spearman correlation coefficient = 0.334, *p* < 0.001), but there was no significant correlation between BMD z-scores and HAZ values (Spearman correlation coefficient = 0.131, *p* = 0.128) (Fig. 2).

### Patients’ follow-up visits to nutrition outpatient clinic

A total of 37 children re-visited our nutrition outpatient clinic after (147 ± 127) days of their last visit. As mentioned in the method part, a WAZ/BMI z-score lower than -2 was defined as acute malnutrition, and a HAZ lower than -2 was defined as chronic malnutrition. The WAZ/BMIZ value of these patients at the follow-up visit was significantly improved compared to that at their first visit (-0.8 versus -1.14, *p* = 0.004). The HAZ value of these patients at the follow-up visit was not statistically different from that at their first visit (-1.52 versus -1.51, *p* = 0.474).

The BMD z-score of the re-visited patients was significantly improved from that of patients at their first visit (-0.10 versus -0.55, *p* = 0.001).

### Serological examination

At patients’ first clinic visit, their median serum 1,25-(OH)_2_-VitD3 level was 23.21 (19.65, 29.11) ng/mL (normal range: > 30 ng/mL), median serum calcium concentration was 1.70 (1.60, 1.75) mmol/L (normal range: 2.23–2.8 mmol/L), median serum ALT level was 35.00 (26.00, 53.00) IU/L (normal range: 0-75 IU/L), and median serum AKP level was 256.00 (192.00, 334.75) IU/L (normal range: 40–150 IU/L). At patients’ follow-up visit, their median serum ALT level was 43.50 (28.00, 67.25) IU/L, and their median AKP level was 305.00 (240.50, 366.25) IU/L.

## Discussion

### Malnutrition in patients undergoing liver transplantation (before and after)

Liver transplantation is the preferred treatment for patients with end-stage liver disease. Patients’ nutritional status is one of the independent risk factors contributing to mortality rates after liver transplantation. M. Tessitore et al. [16] undertook a narrative review of both recent and relevant older literature, published during the last 20 years, for studies linking nutrition to pediatric chronic cholestasis. The collected data confirm that malnutrition and failure to thrive are associated with increased risks of morbidity and mortality, and they also affect the outcomes of liver transplantation, including long-term survival. Few studies reported the nutritional status of cholestatic children, and typically include small cohorts. V. Fouqet et al. published a study in 2005, and reported that the prevalence of malnutrition of 280 children diagnosed biliary atresia was 49% [17]. We assessed the nutritional status of all the patients who visited our nutrition outpatient clinic. As the results of this study, 31.9% of the patients who visited our nutrition outpatient clinic had acute malnutrition, and 38.4% of them had chronic malnutrition. After classifying the patients according to the time-line of liver transplantation, it was found that in the patients before liver transplantation, the proportion of acute malnourished infants was higher than that of chronic malnourished infants (53.8% vs 30.8%). In the patients more than 100 days after liver transplantation, the proportion of chronic malnourished infants was higher than that of acute malnourished infants (24.4% vs 42.2%). It might be that with the improvement of liver status, the amount of oral food intake has been improved, followed by the improvement of weight. However, height can be affected by some different factors. For example, nutrition status and the postoperative drugs (such as corticosteroid therapy) which could result in the slow recovery of height. Studies reported that linear growth improves after liver transplantation, yet catch-up growth is greatly impacted by pre- and post-liver transplantation factors. A multicenter study of 892 children who survived beyond the first year post-liver transplantation showed that transplant recipients are shorter than expected based on mid-parental heights [18]. Independent factors associated with shorter stature were pretransplant linear growth impairment, metabolic disease as an indication for liver transplantation, retransplants, and long-term use of corticosteroids post-liver transplantation.

### The level of 1,25-(OH)_2_-D3 and bone mineral density of patients undergoing liver transplantation (before and after)

Osteodystrophy affects both children and adults with chronic liver disease. Liver transplant survivors are recognized to have increased incidence of fractures and osteopenia even at long-term review [19]. Reduced bone mineral density (BMD) within the first 3–4 months after transplantation has been reported in both adults and children. A study has reported that malnutrition exerts a negative impact on BMD in non-cirrhotic individuals [20]. Meanwhile, vitamin D is one of the fat-soluble vitamins, and the active form of vitamin D is known to impact bone function. The prevalence of osteoporosis before liver transplantation is reported to be between 12 and 55%. Most patients already had bone mass loss before liver transplant, which would lead to bone pain, growth retardation, and even bone fracture. Bone mass loss is particularly common in patients within one year after liver transplantation [21–24]. In the early stage after liver transplantation, because of the use of high-dose glucocorticoids and other immunosuppressive drugs, the loss of patients’ bone mass was rapid, particularly the loss of bone mass in the spine and proximal femur.

In this study, we used DXA to measure the BMD in liver transplant patients. It is known to be convenient, safe, noninvasive, accurate, and precise. Another key advantage of DXA is the low radiation exposure. Almost all pediatric patients were cooperative to complete the measurement. 69.6% of the patients had a BMD z-value < 0. We also assessed patients’ serum vitamin D levels and found that 89% of patients had vitamin D levels lower than normal.

In a retrospective study conducted by Xuguang Zhang et al., 55,925 children’s serum samples were collected to study the serum vitD levels. They found that the overall rate of hypovitaminosis D of 65.60% [25] was similar to our study results. We further analyzed the correlation between BMD and nutritional status. As a result, the decrease of BMD was significantly correlated with the index of acute malnutrition (WAZ/BMIZ), suggesting that the daily intake of nutrients has a significant impact on nutritional status of bones in pediatric patients. Our nutrition outpatient clinic provided nutritional guidance and recommended a 400–800 IU/d vitD supplementation. The BMD was significantly improved when the patients re-visited our nutrition clinic.

### Benefits of nutritional intervention to nutritional status during perioperative period of liver transplantation

In 2017, the Nutrients Magazine published a review article on suggestions and literature review of the nutritional status assessment and nutrition care plan management for perioperative liver transplantation patients [26]. It was suggested that the management of liver transplant patients should include a comprehensive nutritional assessment. Adequate nutritional support should be given at all stages of liver transplantation. Oral nutrition was the primary choice, and nutritional formulas, tube feeding and other modes of nutrition support could be selected as needed to achieve the goal of calorie intake. Due to the higher requirements for nutritional intake of pediatric patients during growth and development stage, scientific feeding guidance and management was critical to reduce the incidence of malnourishment, thus affecting patients’ long-term prognosis [27].

Useful literature on the nutritional management of children undergoing liver transplantation is limited [16]. After liver transplantation, weight gain seems to recover completely, despite previous malnutrition.

After we provided nutritional guidance and individualized diets for liver transplant patients, the nutritional status of the patients in their follow-up visit was significantly improved from that in their first visit, where the difference of WAZ/BMIZ-values had a statistical significance.

In this study, 69.6% of the liver transplant children had a low BMD when visited our nutrition outpatient clinic for the first time, and their parents didn’t have enough knowledge on vitD supplementation. Through the nutritional guidance given at our nutrition clinic, the BMD of the follow-up children was significantly improved than that of the first-visit ones (*p* = 0.001). It is suggested that liver transplant patients should be followed up not only in the transplantation outpatient clinic, but also in the nutrition outpatient clinic. In nutrition clinic, patients’ nutrition related problems could be observed in time, and scientific guidance of nutrients supplementation could be given to them, so as to improve their nutritional status including that of their bones. Ultimately, an improvement of the quality of life and the prognosis of patients are expected.

### Limitations

Our study had several limitations. First, our sample size was relatively small. Second, we could not know whether patients’ nutritional status was improved after structured nutritional intervention due to our high loss to follow-up, short duration and low frequency of follow-up visits. Future studies should focus on more and longer follow-up visits to assess if there is improvement in nutritional status and bone health status of infants undergoing liver transplantation after receiving standardized nutritional intervention.

## Conclusion

There were different rates of malnutrition before and after liver transplantation. At the same time, BMD Z-score and serum vitamin D level of patients decreased. There was a significant correlation between BMD z-scores and WAZ/BMIZ values. Professional nutritionists and dietitians are needed to conduct nutritional assessment, and to provide individualized nutritional guidance plus vitD supplementation suggestions for liver transplant pediatric patients. The ultimate goal of improving patients’ nutritional status including that of their bones will be achieved then.

## Data Availability

The datasets used and analyzed in the current study are available from the corresponding author on reasonable request (Li Hong, hongliscmc@163.com).

## Abbreviations

BMD: Bone mineral density
DXA: Dual energy X-ray absorptiometry
HAZs: Height-for-age z-scores
WAZs: Weight-for-age z-scores
BMIZs: BMI-for-age z-scores
LT: Liver transplantation
